# Timely outpatient follow-up and readmission risk in schizophrenia

**DOI:** 10.3389/fpsyt.2026.1922379

**Published:** 2026-09-30

**Authors:** Ji Woon Jeong, Hyeyoung Yoo, Hyeung-Keun Park

**Affiliations:** 1 Jeju Institute of Public Health and Health Policy, Jeju National University Hospital, Jeju, Republic of Korea; 2 Department of Health Policy and Management, College of Medicine, Jeju National University, Jeju, Republic of Korea; 3 Department of Preventive Medicine, Jeju National University Hospital, Jeju, Republic of Korea

**Keywords:** cohort study, outpatient follow-up, readmission, schizophrenia, survival analysis

## Abstract

Timely outpatient follow-up after psychiatric hospitalization has been proposed as a key strategy to reduce readmission; however, evidence in patients with first-admission schizophrenia remains limited. Using the Korean National Health Insurance Claims Database, we conducted a nationwide retrospective cohort study of 1,961 patients with first-admission schizophrenia, defined as having no psychiatric service utilization during the preceding three years. We examined whether outpatient follow-up within 30 days after discharge was associated with the risk of readmission within 365 days. Adjusted Cox proportional hazards models were used to estimate hazard ratios across multiple cumulative follow-up periods (60, 90, 120, 150, 180, and 365 days), controlling for sociodemographic and clinical factors. Kaplan–Meier analysis was used to assess differences in readmission-free survival. Among the study population, 53.2% received outpatient follow-up within 30 days after discharge. When evaluated across progressively longer cumulative follow-up periods, outpatient follow-up was associated with a significantly lower risk of readmission at 60 days (aHR = 0.52, 95% CI: 0.29–0.95), 90 days (aHR = 0.55, 95% CI: 0.35–0.88), 120 days (aHR = 0.63, 95% CI: 0.43–0.94), 150 days (aHR = 0.66, 95% CI: 0.46–0.95), and 180 days (aHR = 0.70, 95% CI: 0.51–0.98), but not at 365 days (aHR = 0.79, 95% CI: 0.61–1.02). Kaplan–Meier analysis showed longer readmission-free survival in the follow-up group, although the difference was more pronounced over shorter follow-up durations. Timely outpatient follow-up was associated with reduced readmission risk over shorter cumulative follow-up periods, with attenuation of the association as the follow-up duration increased.

## Introduction

Schizophrenia is a chronic and severe psychiatric disorder characterized by hallucinations, delusions, disorganized thinking, and substantial functional impairment (1). It affects approximately 0.33%–0.75% of the global population, and systematic reviews have reported a median lifetime prevalence of 0.4% (2). The disorder typically emerges in early adulthood and often follows a relapsing course (3–5). Schizophrenia imposes a considerable burden not only on affected individuals, through reduced quality of life and impaired social functioning, but also on healthcare systems and society because of increased medical costs, caregiver burden, and productivity loss (6, 7).

Preventing relapse and rehospitalization has therefore become a major priority in mental health policy (8). This issue is particularly important in Korea, where readmission rates among psychiatric populations remain high. For example, OECD Health at a Glance 2013 reported that the 30-day readmission rate for schizophrenia in South Korea in 2011 was 19.4%, exceeding the OECD average of 12.3% (9, 10). In addition, nationwide data from Korea have shown that readmission rates among patients with schizophrenia spectrum disorders increased over time, reaching 41.03% and 46.8% within 3 and 6 months after discharge, respectively, in 2017 (11). Although readmission is not equivalent to relapse, it is widely used as a measurable indicator of poor post-discharge stabilization and an important outcome in mental health services research.

Timely outpatient follow-up after psychiatric hospitalization has been proposed as a potentially important strategy for reducing readmission. In particular, outpatient follow-up within 30 days of discharge is commonly used as a key quality indicator in mental health care (12–14). However, evidence regarding its association with psychiatric readmission remains mixed. Several studies have reported that outpatient follow-up within 30 days after discharge was associated with a reduced risk of readmission during the subsequent 90 or 180 days (15–17). Similarly, outpatient care received within 180 days after discharge has been associated with a lower risk of readmission (18). In contrast, other studies did not find a significant association between outpatient care and reduced readmission (19, 20). Moreover, much of the existing literature has been based on broader psychiatric populations, leaving limited evidence specific to patients with first-admission schizophrenia.

Previous studies have identified several demographic and clinical factors associated with psychiatric readmission or frequent hospitalization among patients with schizophrenia, including younger age, male sex, substance use, and shorter hospitalization (21–24). Among these factors, a history of prior psychiatric hospitalization has consistently been reported as an important predictor of subsequent readmission (25–29). For example, one study of 805 psychiatric patients found that a greater number of prior hospitalizations was associated with an increased risk of readmission (HR = 1.42, 95% CI 1.22–1.64) (25), while another study in early psychosis reported that prior admissions increased the odds of readmission (OR = 1.29, 95% CI 1.04–1.61) (28). Because prior service use and hospitalization history may reflect illness chronicity and established patterns of care, they may also confound the association between timely outpatient follow-up and readmission. Restricting the sample to patients with first-admission schizophrenia may therefore allow a clearer assessment of this association.

This study aimed to determine whether timely outpatient follow-up was associated with a reduced risk of readmission among patients with first-admission schizophrenia and whether this association varied across progressively longer follow-up periods. We hypothesized that patients who received outpatient follow-up within 30 days after discharge would have a lower risk of readmission than those who did not receive such follow-up.

## Methods

We conducted a nationwide retrospective cohort study using the National Health Insurance Claims Database (NHICD; NHIS-2023-1-753). South Korea operates a universal single-payer health insurance system that covers the entire population, including both National Health Insurance and Medical Aid beneficiaries. The NHICD contains eligibility data, including age, sex, residence, and income level, as well as healthcare utilization claims data on inpatient, outpatient, and emergency department services. Because healthcare records are linked through unique Resident Registration Numbers assigned to all Korean residents, the database enables longitudinal follow-up of healthcare utilization at the individual level across healthcare settings. All personal identifiers are de-identified before data are provided to researchers. First-admission schizophrenia was operationally defined as hospitalization for schizophrenia with no record of psychiatric service utilization—including inpatient, outpatient, or emergency care—during the three years preceding the index admission.

From the database, we identified all patients who were hospitalized for schizophrenia (ICD-10 code F20) between 2015 and 2020 (n = 117,826). Among these, individuals were included if they had an index admission between January 1, 2018, and December 31, 2019, and met the above definition of first-admission schizophrenia (n = 2,810). To account for split claims, hospitalization episodes were constructed by linking admission records separated by 1 day or less.

Patients were then excluded based on the following criteria: (1) missing data on age, sex, or insurance premium level (n = 27); (2) invalid admission episodes (length of stay [LOS] ≤ 0 days or absence of an inpatient fee code) (n = 174); (3) index hospitalization longer than 365 days (n = 429); (4) death during the index hospitalization (n = 18); and (5) readmission or death within 30 days after discharge (n = 201). Patients with an index hospitalization longer than 365 days were excluded because a full 365-day follow-up after discharge could not be assessed. Patients who were readmitted or died within 30 days after discharge were also excluded because they did not have sufficient time to receive outpatient follow-up within the exposure assessment window. After applying all inclusion and exclusion criteria, a total of 1,961 patients remained in the final analytic sample (**Figure 1**).

**Figure 1:**
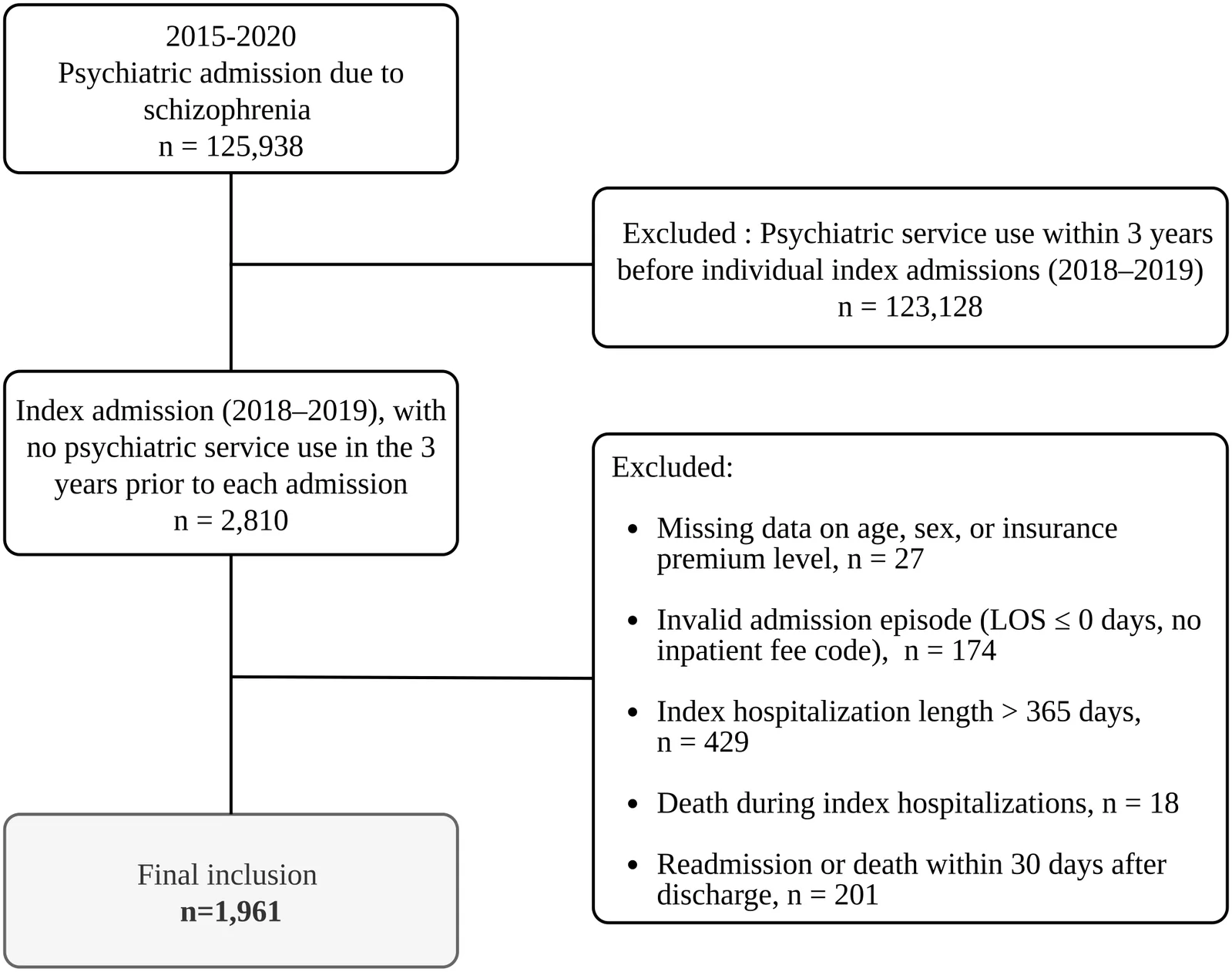
Flowchart of patient selection for the study cohort.

Timely outpatient follow-up was defined as at least one outpatient visit with a diagnosis of schizophrenia within 30 days after discharge from the index hospitalization, regardless of the type of healthcare institution visited. This measure was designed to capture early linkage to outpatient care after discharge rather than the frequency or sustained continuity of subsequent outpatient treatment. In the survival analysis, the event was defined as readmission for schizophrenia (ICD-10 code F20 as the primary diagnosis). Readmission risk was assessed beginning on day 31 after discharge, after completion of the 30-day exposure assessment window. Patients were followed for up to 365 days after discharge. Readmission risk was evaluated at multiple cumulative follow-up periods (60, 90, 120, 150, 180, and 365 days) to examine how the association varied as the duration of follow-up increased.

Covariates included age, sex, income level (categorized as medical aid [Q1] and health insurance premium quintiles [Q2–Q5], with higher quintiles indicating higher income levels), hospital type at index admission (general hospital, hospital, psychiatric hospital, and clinic), and LOS during the index admission (categorized as 1–14, 15–30, 31–90, 91–180, and 181–365 days), as well as psychiatric comorbidity. In South Korea, psychiatric care is provided across different types of healthcare institutions, including general hospitals, hospitals, psychiatric hospitals, and clinics. General hospitals are relatively large medical institutions with at least 100 beds and are required to have multiple specialized departments and medical specialists, whereas hospitals are generally smaller medical institutions with at least 30 beds. These requirements are specified under the Korean Medical Service Act (30). Psychiatric comorbidity was defined as the presence of any additional psychiatric diagnosis (ICD-10 codes F04–F99), excluding schizophrenia (F20), recorded at the time of the index admission.

The distributions of continuous variables were assessed using the Shapiro–Wilk test and visual inspection of histograms and Q–Q plots. Continuous variables with approximately normal distributions were presented as means and standard deviations and compared using independent two-sample t-tests, whereas variables with skewed distributions were presented as medians and interquartile ranges and compared using Wilcoxon rank-sum tests. Categorical variables were presented as frequencies and percentages and compared using chi-square tests. Readmission-free survival was estimated using the Kaplan–Meier method, and differences in survival curves were assessed using the log-rank test. Cox proportional hazards regression models were fitted separately for each cumulative follow-up period (60, 90, 120, 150, 180, and 365 days) to estimate hazard ratios for readmission associated with outpatient follow-up, adjusting for covariates. Patients were censored at the time of death or at the end of each follow-up period if readmission did not occur. All analyses were performed in accordance with National Health Insurance Service (NHIS) security protocols using SAS (SAS Institute, Cary, NC, USA), with statistical significance defined as a two-sided p-value < 0.05. Ethical approval was obtained from the Institutional Review Board of Jeju National University Hospital (IRB No. 2023-03-003-001).

## Results

### Baseline characteristics of the study population

A total of 1,961 patients with first-admission schizophrenia were included in the analysis. Among them, 1,044 patients (53.2%) received at least one outpatient visit within 30 days after discharge, whereas 917 (46.8%) did not. By 365 days after discharge, 1,253 patients (63.9%) had received at least one outpatient visit. Of these patients, 83.3% had their first outpatient visit within 30 days after discharge.

**Table 1** summarizes the baseline characteristics of the study population according to outpatient follow-up within 30 days after discharge. The mean age of the overall sample was 45.9 years (SD, 15.8; range, 13–98 years). Patients who received outpatient follow-up were significantly younger than those who did not (43.0 ± 14.1 vs. 49.2 ± 16.9 years, p < 0.001). Sex also differed significantly between the groups (p < 0.001): female patients accounted for a greater proportion of the follow-up group than of the non-follow-up group (60.5% vs. 48.9%), whereas male patients were more prevalent in the non-follow-up group (51.1% vs. 39.5%).

**Table 1:** Patient characteristics according to outpatient follow-up within 30 days after discharge.

Variable	Total	Outpatient follow-up		p-value
		No	Yes	
Total	1,961	917 (46.8)	1,044 (53.2)	
Age	45.9 ± 15.8	49.2 ± 16.9	43.0 ± 14.1	< 0.001^‡^
Sex				< 0.001^†^
Male	881 (44.9)	469 (51.1)	412 (39.5)	
Female	1,080 (55.1)	448 (48.9)	632 (60.5)	
Income level				< 0.001^†^
Q1	233 (11.9)	151 (16.5)	82 (7.9)	
Q2	551 (28.1)	243 (26.5)	308 (29.5)	
Q3	403 (20.6)	181 (19.7)	222 (21.3)	
Q4	391 (19.9)	177 (19.3)	214 (20.5)	
Q5	383 (19.5)	165 (18.0)	218 (20.9)	
Hospital type				< 0.001^†^
General hospital	272 (13.9)	85 (9.3)	187 (17.9)	
Hospital	956 (48.8)	475 (51.8)	481 (46.1)	
Psychiatric hospital	646 (32.9)	319 (34.8)	327 (31.3)	
Clinic	87 (4.4)	38 (4.1)	49 (4.7)	
Length of stay (days)	47 (19–92)	31 (10–86)	59 (30–99)	< 0.001^§^
Psychiatric comorbidity				0.012^†^
No	766 (39.1)	331 (36.1)	435 (41.7)	
Yes	1,195 (60.9)	586 (63.9)	609 (58.3)	

Data are presented as mean ± standard deviation, median (interquartile range), or n (%), as appropriate.

^†^ Chi-square test; ^‡^Student’s t-test; ^§^Wilcoxon rank-sum test.

Income level was also significantly associated with outpatient follow-up (p < 0.001). Patients in the non-follow-up group were more likely to be in the lowest income category (Q1: 16.5% vs. 7.9%), whereas those in the follow-up group were more likely to be in the higher income categories. Hospital type at index admission differed significantly between the groups (p < 0.001). In particular, patients discharged from general hospitals were more likely to receive outpatient follow-up than those discharged from other facility types (17.9% vs. 9.3%). In contrast, patients discharged from hospitals and psychiatric hospitals were relatively more concentrated in the non-follow-up group.

The median length of stay during the index hospitalization was significantly longer in the follow-up group than in the non-follow-up group (59 days [IQR, 30–99] vs. 31 days [IQR, 10–86], p < 0.001). Psychiatric comorbidity also differed significantly between the groups (p < 0.05), with a higher proportion of psychiatric comorbidity in the non-follow-up group than in the follow-up group (63.9% vs. 58.3%).

### Association between outpatient follow-up and risk of readmission

Overall, outpatient follow-up within 30 days was associated with a lower risk of readmission over shorter cumulative follow-up periods, but this association weakened as the follow-up duration increased (**Table 2**). When evaluated across progressively longer cumulative follow-up periods, outpatient follow-up was associated with a significantly lower risk of readmission at 60 days (aHR = 0.52, 95% CI: 0.29–0.95), 90 days (aHR = 0.55, 95% CI: 0.35–0.88), 120 days (aHR = 0.63, 95% CI: 0.43–0.94), 150 days (aHR = 0.66, 95% CI: 0.46–0.95), and 180 days (aHR = 0.70, 95% CI: 0.51–0.98). However, this association was progressively attenuated as the follow-up duration increased and was no longer statistically significant at 365 days (aHR = 0.79, 95% CI: 0.61–1.02).

**Table 2:** Adjusted Cox proportional hazards models for readmission according to outpatient follow-up within 30 days after discharge.

Variable	60 days	90 days	120 days	150 days	180 days	365 days
Outpatient follow-up						
No	1.00	1.00	1.00	1.00	1.00	1.00
Yes	**0.52 (0.29–0.95)**	**0.55 (0.35–0.88)**	**0.63 (0.43–0.94)**	**0.66 (0.46–0.95)**	**0.70 (0.51–0.98)**	0.79 (0.61–1.02)
Age	1.01 (0.99–1.03)	1.01 (0.99–1.02)	1.01 (0.99–1.02)	1.01 (1.00–1.02)	1.01 (1.00–1.02)	1.00 (1.00–1.01)
Sex						
Male	1.00	1.00	1.00	1.00	1.00	1.00
Female	0.72 (0.42–1.23)	0.80 (0.53–1.22)	0.73 (0.51–1.05)	0.73 (0.52–1.01)	0.80 (0.59–1.08)	0.91 (0.72–1.15)
Income level						
Q1	1.00	1.00	1.00	1.00	1.00	1.00
Q2	0.79 (0.36–1.75)	0.75 (0.41–1.37)	0.76 (0.45–1.30)	0.79 (0.48–1.30)	0.79 (0.49–1.25)	0.82 (0.57–1.19)
Q3	0.85 (0.37–1.99)	0.70 (0.36–1.36)	0.62 (0.34–1.13)	0.72 (0.42–1.25)	0.72 (0.44–1.20)	0.69 (0.46–1.03)
Q4	0.48 (0.18–1.27)	**0.46 (0.22–0.96)**	**0.50 (0.26–0.94)**	0.57 (0.32–1.01)	**0.55 (0.32–0.94)**	**0.53 (0.34–0.81)**
Q5	0.80 (0.33–1.95)	0.75 (0.38–1.49)	0.72 (0.40–1.32)	0.74 (0.42–1.30)	0.82 (0.49–1.37)	0.76 (0.50–1.14)
Hospital type						
General hospital	1.00	1.00	1.00	1.00	1.00	1.00
Hospital	**8.36 (1.13–61.78)**	**4.84 (1.50–15.65)**	**2.60 (1.18–5.73)**	1.77 (0.95–3.29)	1.57 (0.91–2.70)	1.44 (0.96–2.17)
Psychiatric hospital	5.72 (0.75–43.59)	**3.45 (1.04–11.45)**	1.95 (0.86–4.44)	1.32 (0.69–2.54)	1.20 (0.67–2.13)	1.13 (0.73–1.74)
Clinic	**14.86 (1.73–127.76)**	**5.06 (1.20–21.27)**	**4.06 (1.50–10.97)**	**2.60 (1.12–6.04)**	2.15 (0.99–4.65)	1.74 (0.94–3.22)
Length of stay (days)						
1–14	1.00	1.00	1.00	1.00	1.00	1.00
15–30	1.23 (0.56–2.73)	1.13 (0.60–2.13)	1.34 (0.76–2.36)	1.17 (0.69–1.99)	1.02 (0.62–1.67)	1.07 (0.72–1.59)
31–90	0.71 (0.33–1.54)	0.84 (0.47–1.51)	1.09 (0.65–1.84)	1.16 (0.73–1.85)	1.13 (0.74–1.73)	1.18 (0.84–1.67)
91–180	0.85 (0.35–2.04)	0.80 (0.40–1.61)	0.69 (0.35–1.35)	0.69 (0.37–1.28)	0.69 (0.40–1.21)	0.91 (0.60–1.39)
181–365	1.27 (0.51–3.15)	1.25 (0.61–2.56)	1.34 (0.70–2.57)	1.34 (0.74–2.43)	1.33 (0.77–2.30)	**1.54 (1.00–2.37)**
Psychiatric comorbidity						
No	1.00	1.00	1.00	1.00	1.00	1.00
Yes	1.16 (0.66–2.02)	1.05 (0.69–1.62)	0.93 (0.64–1.35)	1.02 (0.72–1.43)	1.06 (0.77–1.44)	1.10 (0.86–1.40)

Models were adjusted for age, sex, income level, hospital type, length of stay, and psychiatric comorbidity.

aHR, adjusted hazard ratio; CI, confidence interval. Bold values indicate statistically significant associations (p < 0.05).

Kaplan–Meier survival analysis also showed longer readmission-free survival among patients who received outpatient follow-up within 30 days after discharge (**Figure 2**). The survival curves remained separated throughout follow-up, with a larger difference observed over shorter follow-up durations (log-rank p = 0.0104). These findings are consistent with the Cox regression results, which showed a significantly lower risk of readmission during the earlier follow-up periods and no statistically significant effect at 365 days.

**Figure 2:**
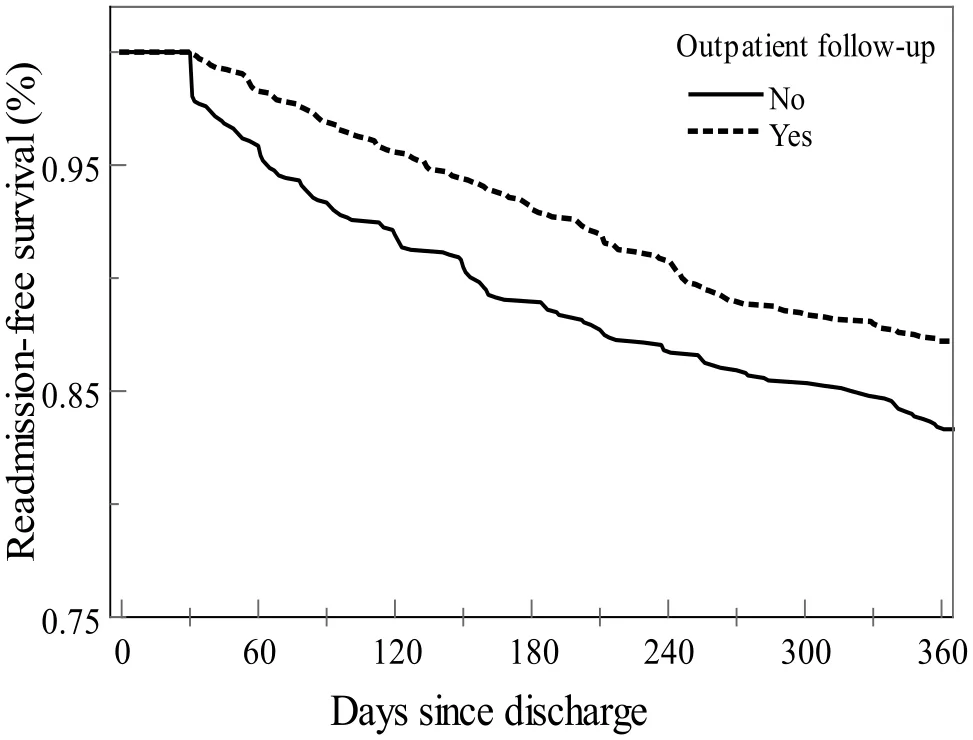
Kaplan–Meier curves for readmission-free survival according to outpatient follow-up within 30 days after discharge.

### Associations of covariates with the risk of readmission

As shown in **Table 2**, income level and hospital type were significantly associated with readmission risk at selected cumulative follow-up periods, whereas sex, length of stay, and psychiatric comorbidity were not significantly associated with readmission across the study period. With respect to income level, compared with the lowest income group (Q1), patients in the fourth income quintile (Q4) had a significantly lower risk of readmission at 90 days (aHR = 0.46, 95% CI: 0.22–0.96), 120 days (aHR = 0.50, 95% CI: 0.26–0.94), 180 days (aHR = 0.55, 95% CI: 0.32–0.94), and 365 days (aHR = 0.53, 95% CI: 0.34–0.81). No statistically significant associations were observed for the other income categories.

Hospital type was also associated with readmission risk, particularly during the earlier follow-up periods. Compared with general hospitals, discharge from hospitals was associated with a higher risk of readmission at 60 days (aHR = 8.36, 95% CI: 1.13–61.78), 90 days (aHR = 4.84, 95% CI: 1.50–15.65), and 120 days (aHR = 2.60, 95% CI: 1.18–5.73). Discharge from psychiatric hospitals was associated with a higher risk at 90 days (aHR = 3.45, 95% CI: 1.04–11.45), and discharge from clinics was associated with a higher risk at 60 days (aHR = 14.86, 95% CI: 1.73–127.76), 90 days (aHR = 5.06, 95% CI: 1.20–21.27), 120 days (aHR = 4.06, 95% CI: 1.50–10.97), and 150 days (aHR = 2.60, 95% CI: 1.12–6.04). However, these associations were attenuated and were no longer statistically significant at later follow-up intervals.

Age was not significantly associated with readmission risk across all follow-up intervals. Female sex was associated with lower hazard estimates than male sex, but this did not reach statistical significance. Length of stay was not significantly associated with readmission at most follow-up intervals, although the highest category showed a marginal estimate at 365 days (aHR = 1.54, 95% CI: 1.00–2.37).

## Discussion

The observed association between timely outpatient follow-up and reduced readmission risk is consistent with previous studies reporting that timely post-discharge care is associated with improved outcomes in patients with severe mental illness. For example, a population-based study in Canada reported that outpatient follow-up within 30 days was associated with a 15–19% reduction in readmission risk (16), while similar associations have been observed across diverse settings, including the United States and Israel (15, 18). Timely follow-up may facilitate continuity of care, support medication adherence, and enable timely reassessment of patients’ clinical status, as well as early detection of symptom exacerbation (16, 31, 32).

At the same time, the attenuation of the association observed in this study as the follow-up duration increased is consistent with prior findings (31, 33). Specifically, previous studies have demonstrated that the protective effect of outpatient visits decreases as the duration of follow-up increases and may no longer be evident at longer follow-up durations (31). These findings suggest that timely outpatient follow-up within 30 days alone may be insufficient to sustain long-term stability. Future studies are needed to determine whether sustained engagement with outpatient services and continuity of care are associated with longer-term reductions in readmission risk.

In addition to outpatient follow-up, income level was associated with readmission risk in this study. Patients in the fourth income quintile showed a consistently lower risk of readmission across several cumulative follow-up periods compared with those in the lowest income group. This finding is consistent with previous research suggesting that socioeconomic factors are associated with outcomes following psychiatric hospitalization (32, 33). Individuals with lower socioeconomic status may face a range of social and environmental challenges, including limited family and social support, unemployment, and housing instability, which may adversely affect recovery and increase the risk of readmission. The absence of a consistent gradient across all income levels suggests that the relationship between income and readmission is likely shaped by multiple pathways beyond income alone.

Hospital type at index admission was also associated with readmission risk, particularly at shorter follow-up durations. Patients discharged from hospitals, psychiatric hospitals, and clinics showed a higher risk of readmission compared with those discharged from general hospitals, although these differences became less pronounced as the follow-up duration increased. A national assessment by the Health Insurance Review and Assessment Service also reported higher 30-day readmission rates after discharge from psychiatric hospitals and clinics than from general hospitals (34). Similarly, a previous Korean nationwide claims-based study reported a higher risk of readmission among patients discharged from hospitals and clinics than among those discharged from general hospitals (31). The authors suggested that these differences may reflect differences in patient case-mix across hospital types rather than differences in treatment quality (31). Because our claims data did not capture detailed clinical characteristics such as symptom severity or functional status, we could not determine the extent to which differences in patient case-mix contributed to the observed associations with hospital type. Therefore, the observed differences in readmission risk across hospital types should be interpreted cautiously. In addition, the estimates for clinics had wide confidence intervals, particularly during the shorter follow-up periods, indicating limited precision.

In contrast, age, sex, length of stay, and psychiatric comorbidity were not significantly associated with readmission risk in this study. Although previous studies have identified younger age, male sex, and psychiatric comorbidities as risk factors for readmission, the association with length of stay has shown inconsistent results across studies (25, 31, 32), with some reporting increased risk with shorter stays and others with longer stays. The lack of significant findings in the present study may be related to the specific characteristics of the study population, which was restricted to patients with first-admission schizophrenia.

The mean length of stay during the index hospitalization was 70.3 days (SD, 71.6), with a median of 47 days (IQR, 19–92). Direct comparison with national Korean data is limited because available estimates are based on different study populations and measures of hospitalization duration. Nevertheless, prolonged psychiatric hospitalization has historically been reported in Korea (35). For context, a median hospitalization duration of 91 days was reported among discharged Medical Aid patients with schizophrenia in a 2019 national evaluation (34). Because our claims data did not include symptom severity, functional status, or other clinical determinants of hospitalization duration, the specific factors underlying the observed length of stay could not be determined.

This study has several strengths. By restricting the analysis to patients with no psychiatric service utilization during the three years preceding the index admission, we reduced the potential influence of recent psychiatric treatment and hospitalization history, thereby allowing a more focused assessment of early post-discharge factors. In addition, the use of a nationwide claims database enhances the generalizability of the findings. The analysis across multiple cumulative follow-up periods also provides insight into how the association between outpatient follow-up and readmission changes as the follow-up duration increases.

Several limitations should be considered. First, first-admission schizophrenia was operationally defined using a three-year washout period, which does not necessarily identify first-ever psychiatric hospitalizations. Although this approach is consistent with prior claims-based studies (36–38), some degree of misclassification is possible, as individuals with prior illness history may relapse after a prolonged period of clinical stability and be reclassified as incident cases in administrative data. Longitudinal studies have shown that relapse can occur beyond the initial years following onset (8, 39). Nevertheless, given that most relapses occur within the early phase of illness (8, 40), the use of a three-year washout period remains a pragmatic approach to reduce the influence of recent psychiatric service use while maintaining sufficient sample size.

Second, outpatient follow-up was defined as at least one visit within 30 days, without accounting for the frequency or continuity of care. This may limit the ability to fully capture the intensity and sustained engagement of outpatient treatment, which could differentially influence readmission risk. Future research should incorporate measures of visit frequency and continuity of care to better elucidate these relationships.

Finally, several clinically relevant factors could not be assessed in this study. Duration of illness could not be reliably determined from claims data because the onset of schizophrenia may precede the first recorded healthcare encounter. In addition, the customized dataset did not include the detailed prescription information required to assess antipsychotic dose and polypharmacy. Substance use, which has been associated with repeated hospitalization among patients with schizophrenia, was also not assessed in this study (24). Other unmeasured factors, such as symptom severity, treatment adherence, and social support, may influence both outpatient service use and readmission risk. Therefore, residual confounding due to unmeasured clinical and treatment-related factors cannot be ruled out, and causal inference remains limited.

## Conclusion

In conclusion, outpatient follow-up within 30 days after discharge was associated with a lower risk of readmission among patients with first-admission schizophrenia, particularly over shorter follow-up durations. These findings suggest that timely outpatient engagement may be an important component of post-discharge care, although its association with readmission risk appears to attenuate as the follow-up duration increases.

## Data Availability

The data analyzed in this study is subject to the following licenses/restrictions: Access to the National Health Insurance Claims Database is restricted by the National Health Insurance Service of Korea. Data are not publicly available and can only be accessed by approved researchers following a formal application and review process. Requests to access these datasets should be directed to National Health Insurance Service (NHIS), Republic of Korea. Requests for access to the National Health Insurance Claims Database can be made through the National Health Insurance Sharing Service (NHISS): https://nhiss.nhis.or.kr.
